# Downregulation of the CCK-B Receptor in Pancreatic Stellate Cells Blocks Molecular Proliferative Pathways and Increases Apoptosis to Decrease Pancreatic Cancer Growth In Vitro

**DOI:** 10.3390/ijms262311699

**Published:** 2025-12-03

**Authors:** Miranda Ortega, Eri Agena, Wenqiang Chen, Hong Cao, Sona Vasudevan, Narayan Shivapurkar, Mariaelena Pierobon, Jill P. Smith

**Affiliations:** 1 Departments of Biochemistry and Molecular & Cellular Biology, Georgetown University, Washington, DC 20007, USA; 2 Department of Medicine, Georgetown University, Washington, DC 20007, USA; 3 Center for Applied Proteomics and Molecular Medicine, School of Systems Biology, George Mason University, Manassas, VA 20110, USA

**Keywords:** cancer-associated fibroblast, tumor microenvironment, molecular signaling pathways

## Abstract

Pancreatic cancer is characterized by an extensive fibrotic stroma largely driven by activated pancreatic stellate cells (PSCs)/fibroblasts, which also function to support tumor growth and metastasis. Cholecystokinin-B receptors (CCK-BRs) are expressed on pancreatic stellate cells (PSCs) and have emerged as a key regulator of PSC activation and tumor-stromal interactions. We hypothesized that disrupting CCK-BR function shifts PSCs to a more quiescent phenotype and reduces their pro-fibrotic and tumor-supportive activity to decrease growth of pancreatic cancer. Murine PSCs were genetically engineered with CRISPR-*Cas9* to knockout the CCK-BR. In a series of experiments, the role of the CCK-BR expression was evaluated on cell migration, proliferation, differentially expressed genes, molecular signaling pathways, and in co-culture with murine pancreatic cancer epithelial cells. Next, primary human pancreatic stellate cells were treated with proglumide, a CCK-BR antagonist, to study the effects of pharmacologic blockade of the CCK-BR on cellular signaling and proliferative pathways by RNA sequencing. Knockout of the CCK-BR led to significant decreases in PSC activation and the ability to stimulate growth of pancreatic cancer cells in co-culture. Both genetic knockdown and pharmacologic blockade of the CCK-BR downregulated genes implicated in fibrosis, proliferation, fibroblast activation, and tumorigenesis, while genes implicated in apoptosis and tumor suppression were upregulated. Flow cytometry showed increased apoptosis markers in CCK-BR-knockout cells compared to controls. These experiments combine transcriptomic profiling with functional validation to provide a comprehensive analysis of how targeting CCK-BR interrupts the cross-communication between cancer cells and fibroblasts. Blockade or downregulation of the CCK-BR on pancreatic fibroblasts may provide a strategy to disrupt oncogenic signaling pathways and reprogram the tumor microenvironment.

## 1. Introduction

Pancreatic ductal adenocarcinoma (PDAC) is one of the most aggressive cancer types, with a 5-year survival rate of about 12–13% [1]. A dense fibrosis characteristically develops around pancreatic tumors, physically blocking immune cells and chemotherapies [2]. This fibrosis is primarily produced by activated pancreatic stellate cells (PSCs) or activated fibroblasts. Quiescent PSCs are the resident fibroblasts that exhibit plasticity, and when activated, they exhibit a phenotypic change characterized by proliferation, migration, extracellular matrix (ECM) remodeling, and immunosuppression, among other factors [1,2,3]. The PSC plays a central role in the pancreas stroma and the development of the extracellular matrix. In addition to the PSC, the pancreatic cancer microenvironment also harbors cancer-associated fibroblasts (CAFs) that have a heterogeneous population [2], including inflammatory CAFs, myofibroblast CAFs, and even antigen-presenting CAFs [2,4]. CAFs may be derived from multiple cellular origins, including from activated resident PSCs, but also from bone marrow-derived mesenchymal stem cells, and epithelial-to-mesenchymal transition (EMT)-derived fibroblasts [5]. Hence, activated resident PSCs are a subtype of CAFs.

CAFs cross-communicate with cancer epithelial cells in the pancreatic tumor microenvironment through paracrine mechanisms [6] and by the release of PSC-derived extracellular vesicles [7] containing cargo such as microRNAs that stimulate the growth of pancreatic cancer [8]. The interaction between cancer cells and fibroblasts is reciprocal, and cancer cells also influence the activity of the fibroblasts. One mechanism of communication is by the release of inflammatory cytokines such as transforming growth factor-beta (TGF-β), interleukin-1β (IL-1β), interleukin-6 (IL-6), chemokine (C-X-C motif) ligand 12 (CXCL12), and leukemia inhibitory factor (LIF) [9,10]. TGF is the most well-studied cytokine that activates fibroblasts [11] that is involved in epithelial-to-mesenchymal transition and metastases. The CAFs play an important role in the progression of pancreatic cancer, increasing tumor growth and promoting metastases [12].

Activated PSCs release collagen, fibronectin, and matrix-degrading enzymes such as matrix metalloproteinases (MMPs) and their inhibitors, tissue inhibitors of matrix metalloproteinases (TIMPs), which are released into the surrounding extracellular matrix [13]. MMPs are zinc-dependent proteases that play an important role in cancer progression by contributing not only to inflammatory cascades but also to ECM degradation and tissue remodeling, resulting in cancer cell invasion, proliferation, and metastatic progression [14,15]. The remodeling by MMPs promotes the spread of cancer cells by reprogramming cancer cells through a process called epithelial–mesenchymal transition (EMT) [16]. Since PSCs are also important for normal physiologic tissue remodeling and wound healing, strategies are being sought to induce plasticity of the fibroblasts to render them quiescent rather than eliminate them.

Cholecystokinin A and B receptors (CCK-BRs) have been identified on PSCs [3]. Upon activation of the CCK receptors, the cells change from a quiescent phenotype, resulting in proliferation, migration, and collagen production. We previously showed that the CCK-BR on the PSC was responsible for these phenotypic changes, not the CCK-A receptor [17]. Furthermore, treatment of the PSCs with a CCK receptor antagonist, proglumide, induced plasticity of the activated fibroblasts, making them more quiescent. Blockade of the CCK-BR with proglumide also decreased collagen production and hydroxyproline (a major component of the extracellular matrix) [17]. In vivo, pancreatic cancers grown in mice exhibited significantly decreased intratumoral fibrosis when treated with proglumide [18]. This decrease in the desmoplastic fibrotic tissue surrounding the pancreatic cancer epithelial cells allowed for the increased penetration of chemotherapy [18] and immune checkpoint inhibitors [19,20], which both significantly improved response. The goal of this work is to examine the role of the CCK-BR molecular and protein signaling pathways in activated pancreatic stellate cells and determine how interruption of this pathway can decrease the cancer-PSC cross communication and growth of pancreatic cancer. In this investigation, we examined the effects of genetic editing of the CCK-BR in murine PSC and also pharmacologic blockade of the CCK-BR in human PSC to understand the mechanisms of how interruption of the CCK-BR signaling pathway in PSC may decrease the aggressive nature of pancreatic cancer.

## 2. Results

### 2.1. Effects of CCK-BR Knockout on Migration, Proliferation, and Growth of Pancreatic Cancer

#### 2.1.1. Effects of CCK-BR-KO on Migration of PSCs

**Figure 1:**
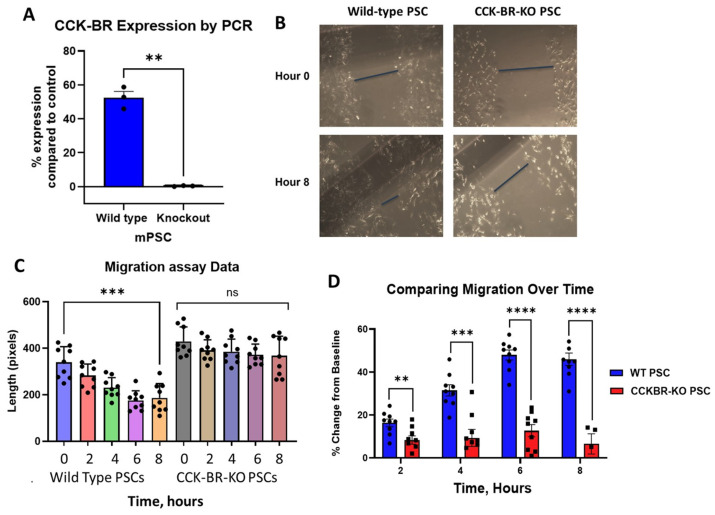
Effects of CCK-BR expression on migration of murine pancreatic cancer cells. (**A**) After CRISPR-*Cas9* knockdown of the CCK-BR, expression of the receptor was measured by qRT-PCR in wild-type and knockdown pancreatic stellate cells. Percent CCK-BR mRNA expression in wild-type PSC is compared to expression in knockout cells. (**B**) Images taken from representative tissue culture wells showing measurement of the scratch width at baseline (0 h) and again after 8 h. (**C**) Scratch width over the course of 8 h, comparing wild-type cells to CCK-BR-KO cells. There is a significant decrease in scratch width between the 0 h and 8 h time points for WT PSCs, but not for CCKBR-KO PSCs. (**D**) Comparisons of percent change in scratch width between WT and CCKBR-KO PSCs. At every time point, WT PSCs had a significantly greater percent change from baseline than CCKBR-KO PSCs. ** *p* < 0.01; *** *p* < 0.005; and **** *p* < 0.001.

We selectively downregulated CCK-BR by CRISPR-*Cas9* technology to understand the role of the CCK-BR expression on pancreatic stellate cell function. Confirmation of decreased CCK-BR mRNA expression was confirmed by qRT-PCR, showing significantly decreased expression compared to wild-type PSCs (Figure 1A). Next, murine wild-type and CCK-BR-KO PSCs were grown to confluency in 12-well plates, and migration of the cells was evaluated by measuring the rate at which adherent cells would close a manually created scratch or gap. Wild-type PSCs migrated in cell culture to close the gap within 8 h (Figure 1B, left). In contrast, the CCK-BR-KO pancreatic stellate cells failed to close the gap over the same period of time (Figure 1B, right). The mean size of the gap was measured over time, and Figure 1C shows the gap size of the wild-type PSCs significantly decreasing over the 8-h time interval, suggesting the wild-type cells are actively migrating. Simultaneously, there was no significant change in the gap size in the CCK-BR-KO cells, implying slowed migration. Figure 1D compares the gap size of the wild-type to the CCK-BR-KO cells for each time point.

#### 2.1.2. Effects of CCK-BR-KO on Proliferation of Pancreatic Stellate Cells and Cancer Cells

**Figure 2:**
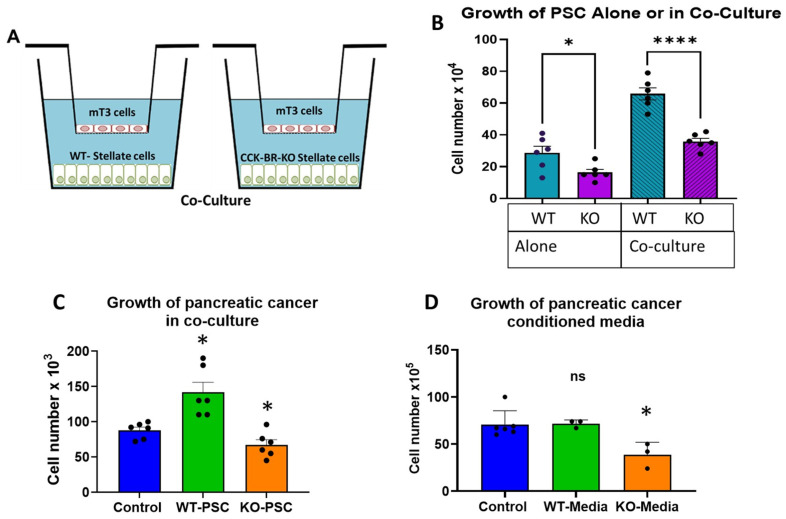
Influence of the CCK-BR expression on PSC growth and in cross-communication with murine mT3 pancreatic cancer cells. (**A**) Schematic of the methods for co-culture experiments with wild-type or CCK-BR-KO PSC grown on the bottom of the 12-well plates in co-culture with murine mT3 PDAC cells grown in the basket insert. (**B**) Growth of wild-type (WT) PSC or CCK-BR-KO (KO) PSC grown in culture alone or co-cultured with PDAC cells. Significantly different with student’s *t*-test * *p* < 0.05 and **** *p* < 0.0001. (**C**) Growth of mT3 pancreatic cancer cells in co-culture transwell plates with wild-type PSCs or CCK-BR-KO PSCs. Significantly different from Welch’s two-tailed *t*-test * *p* < 0.05. (**D**) Effects of conditioned media from wild-type PSC (WT-media) or conditioned media from CCK-BR-KO PSC (KO-media) compared to the growth of mT3 cells in fresh DMEM media (control). * *p* < 0.05; ns = not significant.

To determine whether the CCK-BR expression on PSC influenced the growth of PSC alone or when co-cultured with murine mT3 pancreatic cancer cells using the transwell culture system (Figure 2A), we performed a series of in vitro growth assays. Knockout of the CCK-BR by CRISPR-*Cas9* on murine PSCs slowed the cell growth by 43% (*p* < 0.05) compared to wild-type PSCs that express the CCK-BR (Figure 2B, left). Proliferation of CCK-BR-KO PSCs was also slower than wild-type PSC when co-cultured with pancreatic cancer cells by 46% (Figure 2B, right; *p* < 0.0001). When PSC were co-cultured with the mT3 murine PDAC cancer cells, the number of wild-type PSCs significantly increased by 2.3-fold in number compared to wild-type PSCs grown independently, and this change was statistically significant (Figure 2B; cyan columns; *p* < 0.0001). The increase in PSC proliferation in co-culture with PDAC cells suggests that the cancer cells release growth factors or exosomes into the culture media to stimulate the growth of the PSCs.

In reciprocal, to study how the CCK-BR signaling pathway influences the cross-communication and growth of pancreatic cancer cells, the mT3 cancer cell number was counted after co-culture for 5 days with wild-type or CCK-BR-KO PSC. The mT3 cell number after growth in co-culture with wild-type PSC was 36% greater than mT3 co-cultured with CCK-BR-KO PSC (Figure 2C, green column; *p* < 0.05), suggesting that the CCK-BR expression on PSC is in part responsible for the proliferative effect on PDAC cells in the tumor microenvironment and influences the cross-communication between the stellate cells and the cancer cells. Co-culture of mT3 cells with CCK-BR-KO cells showed a significant decrease in pancreatic cancer cell number compared to control cells without co-culture (Figure 2C, orange column; *p* < 0.05), and this was also less than the number of mT3 cells co-cultured with wild-type PSC.

Conditioned media was collected from both wild-type and CCK-BR PSCs and applied to mT3 pancreatic cancer cells (Figure 2D). Spent media from the wild-type PSC did not increase proliferation of the pancreatic cancer cells compared to mT3 cells grown with fresh media (Figure 2D, green column). However, the number of mT3 cells significantly decreased in vitro when exposed to spent media from the CCK-BR-KO PSC (Figure 2D, orange column; *p* < 0.05). These results support the cross-communication between PSCs and pancreatic cancer epithelial cells. Furthermore, the decreased pancreatic cancer growth in mT3 cells with co-culture or with conditioned media from the knockout PSCs suggests that elimination of the CCK-BR signaling pathway interrupts paracrine mechanisms of communication or extracellular vesicles released from the PSC.

### 2.2. Differentially Expressed Genes by RNA Sequencing

**Figure 3:**
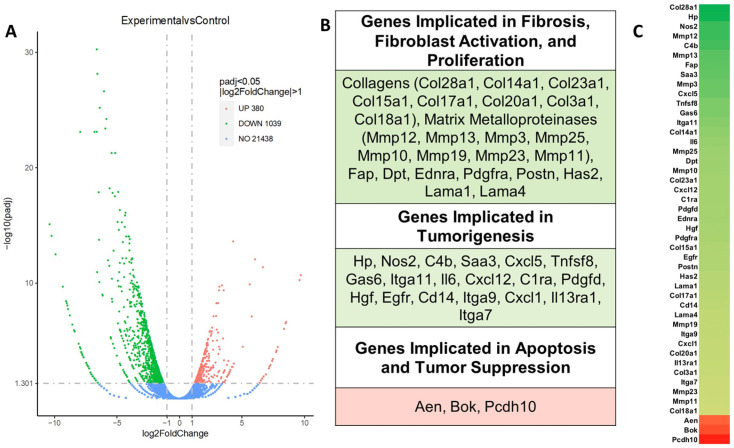
Differentially expressed genes (DEGs) in CCK-BR-KO PSCs compared to WT PSCs, as identified by RNA-sequencing analysis. (**A**) Volcano plot displaying the distribution of DEGs with cutoffs of |Log2FoldChange| > 1 and *p*-adjusted < 0.05. Each dot respresents a gene: green dots are genes that are downregulated and red dots are genes that are upregulated. (**B**) Functional classification of selected DEGs of interest is shown. Genes highlighted in green indicate downregulation, and red-highlighted genes indicate upregulation. (**C**) Heatmap of relevant genes implicated in fibrosis, proliferation, fibroblast activation, tumorigenesis, tumor suppression, and apoptosis of the CCK-BR-KO PSCs.

RNA was extracted from mouse wild-type and CCK-BR-KO pancreatic cancer cells, and samples with RNA integrity numbers (RIN) greater than or equal to 8 were subjected to RNA sequencing to evaluate differentially expressed genes (DEGs). The volcano plot (Figure 3A) revealed that when the CCK-BR was knocked out with gene editing using CRISPR-*Cas9*, many more genes were downregulated (*n* = 1039; green) compared to the number of genes that were upregulated (*n* = 380, red) in comparison to wild-type stellate cells. The majority of the downregulated genes are implicated in fibrosis, fibroblast activation, proliferation, and tumorigenesis (Figure 3B). Of these, the expression of several matrix metalloproteinases was decreased, including *Mmp10*, *Mmp12*, and *Mmp13*; these metalloproteinases have been implicated in epithelial-to-mesenchymal transition, invasion, and metastases in pancreatic cancer [21,22,23]. The top three genes that were upregulated when the CCK-BR was knocked out of PSCs included *Aen*, *Bok*, and *Pcdh10*; these genes function in pancreatic cancer by increasing apoptosis and tumor suppression. *Aen* is a nuclear exonuclease required for p53-dependent apoptosis [24]. *Bok* is a pro-apoptotic BCL-2 member and a main regulator of the intrinsic apoptotic pathways [25]. *Pcdh10*, or Protocadherin-10, is a tumor suppressor gene that is frequently downregulated by promoter methylation in pancreatic cancer cells [26]. A heat map listing the order and expression level of these differentially expressed genes is shown in Figure 3C.

**Figure 4:**
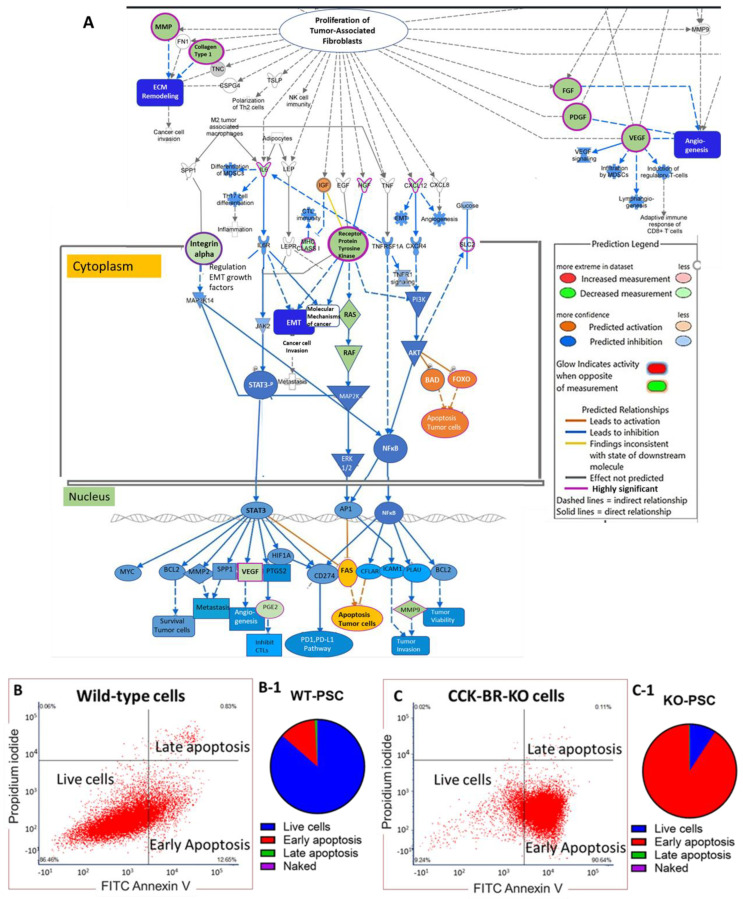
Effects of CCK-BR-KO in murine PSCs on genes affecting the pancreatic tumor microenvironment. (**A**) Pathways involved in proliferation, invasion, and metastasis were downregulated, as demonstrated by the blue or green color. Genes involved in apoptosis were increased and shown by the orange color. Genes outlined in purple are very significant. (**B**) Flow cytometry of wild-type PSCs with apoptosis markers. (**B-1**) Graphical representation of the percentage of wild-type live cells versus cells undergoing apoptosis. (**C**) Flow cytometry of CCK-BR-KO cells with apoptosis markers. (**C-1**) Graphic representation of the percentage of CCK-BR-KO PSC live cells versus cells undergoing apoptosis.

The protein-coding DEGs from the RNA sequencing of the murine PSCs were analyzed using the Ingenuity Pathway Analysis software (version Spring Release, Q1 2025), and confirmed that CRISPR-knockout of the CCK-BR in murine PSCs altered the tumor microenvironment. The downregulated genes (in blue) are involved with pancreatic cancer invasion, metastasis, epithelial-to-mesenchymal transition, and proliferation of cancer-associated fibroblasts (Figure 4A). The upregulated genes (in orange) are involved in apoptosis (Figure 4A).

### 2.3. CCK-BR-KO Increases Markers of Apoptosis on PSCs

To confirm the upregulation of genes involved with apoptosis by RNA sequencing, the PSCs were analyzed for markers of apoptosis by flow cytometry. After five days in culture, wild-type PSCs and CCK-BR-KO PSCs were stained with propidium iodide and Annexin V and subjected to flow cytometry to determine if knockout of the CCK-BR in PSCs increased markers of apoptosis. Figure 4B shows the histogram results of the wild-type PSC with 86.46% of the cells alive, and 12.65% of the cells undergoing early apoptosis. To the right is a graphical display of the percentage of wild-type cells from each quadrant of the histogram. Figure 4C shows a histogram of the flow cytometry of the CCK-BR-KO PSC. In contrast, only 9.24% of the cells are alive after 5 days, and 90.64% of the cells are undergoing early apoptosis. The flow cytometry results support the findings of the RNA sequencing in that knockout of the CCK-BR induces apoptosis in PSCs.

### 2.4. Evaluation of Differentially Expressed Proteins by Reverse Phase Protein Array (RPPA) and Western Blot Analysis

**Figure 5:**
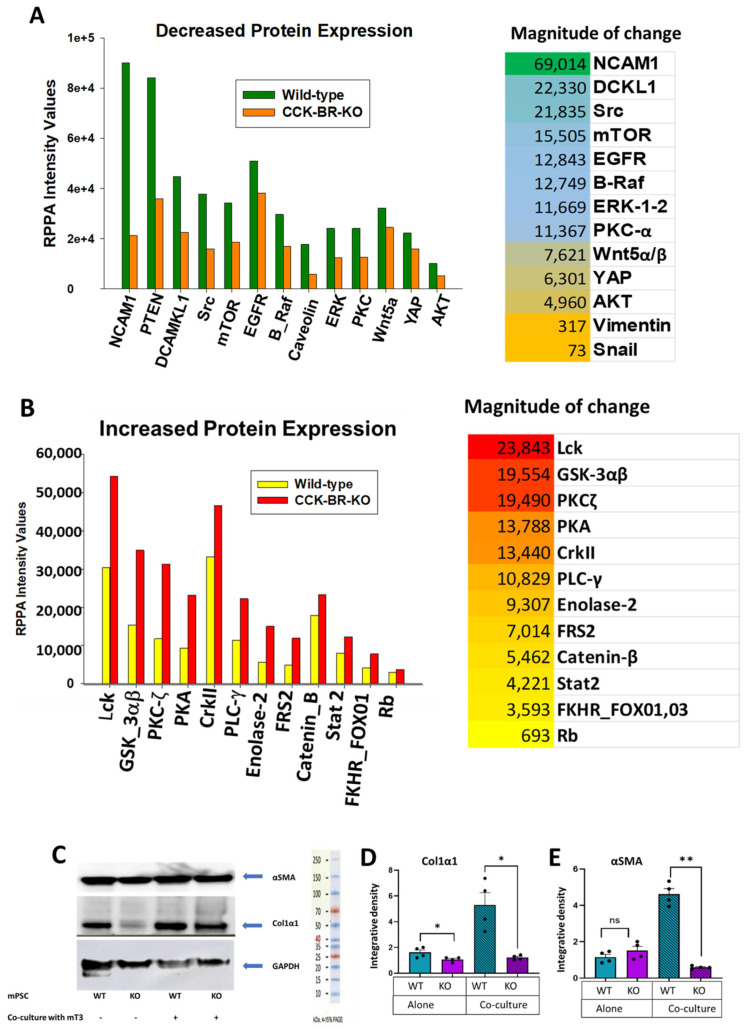
Effect of CCK-BR-KO on murine PSC on protein signaling pathways involved in the pancreatic tumor microenvironment. (**A**) Expression of key target proteins in the pancreatic cancer signaling pathway, analyzed by Reverse Phase Protein Array (RPPA), that are downregulated when the CCK-BR is decreased by CRISPR in murine pancreatic stellate cells. (**B**) Selective stellate cell proteins that increase by RPPA with CCK-BR-KO. (**C**) Colagen1α1 and αSMA protein expression by western blot of wild-type and CCK-BR-KO PSCs grown alone or co-cultured with mT3 pancreatic cancer cells, with GAPDH as the normalizer. The protein ladder marker is shown to the right. (**D**) Densitometry of the western blot shows collagen1α1 protein expression in wild-type or CCK-BR-KO PSCs cultured alone or co-cultured with mT3 pancreatic cancer cells; * *p* < 0.05. (**E**) Densitometry of the western blot for αSMA protein expression in wild-type or CCK-BR-KO PSCs cultured alone or co-cultured with mT3 pancreatic cancer cells. WT: wild-type; KO: knockout; mPSC: mouse pancreatic stellate cells * *p* < 0.05; ** *p* < 0.01; ns = not significant.

Pancreatic stellate cell lysates in triplicate were pooled and analyzed by RPPA to study protein signaling pathways altered by CRISPR downregulation of the CCK-BR in mouse PSC compared to wild-type cells. The top 13 proteins that were significantly decreased by RPPA analysis in the PSC by downregulating CCK-BR are shown in Figure 5A, with the corresponding magnitude of change in RPPA intensity values to the right. NCAM-1, or neural cell adhesion molecule, exhibited the greatest change among the downregulated signaling proteins [27]. Another protein that was significantly decreased with CCK-BR-KO in PSCs was doublecortin-like kinase 1 (DCLK1), a key regulator of fibrosis in several organs by controlling pro-inflammatory and pro-fibrotic signaling pathways [28]. Other signaling proteins that were decreased in CCK-BR-KO PSC compared to wild-type cells included those involved in proliferation (i.e., src, mTOR, AKT, EGFR, ERK) and those involved in epithelial-to-mesenchymal transition (EMT) (i.e., Wnt, vimentin, snail).

Analysis by RPPA also showed that knockout of the CCK-BR in PSC increased RPPA intensity values of several key proteins involved in decreasing fibrosis (Figure 5B). Phosphorylation of the Lck kinase (or lymphocyte tyrosine kinase) at tyrosine residue 505 (Y505) is an inhibitory signal that is functionally linked to fibrosis, and its phosphorylation at Y505 serves as a brake on T-cell activation [29]. Glycogen synthase kinase-3 (GSK3αβ) is in the family of Ser/Thr kinases and has multifaceted functions; phosphorylation at these serine sites inhibits the activity of both the alpha and beta isoforms of the enzyme, resulting in inhibition of fibrosis [30]. Protein Kinase C-zeta (PKCζ) exerts its suppressing effect on ERK activation and proliferation in fibroblasts primarily through interactions that involve MAP kinase phosphatase-1 (MKP-1) [31]. Other phosphorylated proteins increased by CCK-BR-KO in PSC that exert antifibrotic effects include protein kinase A (PKA) [32] and STAT2 [33]. High levels of phosphorylated Catenin-β at the S33, S37, and T41 sites mean the destruction complex is active, preventing Catenin-β accumulation and suppressing cancer-promoting gene transcription [34]. Another tumor suppressor protein that is upregulated in PSCs when CCK-BR is knocked out includes the retinoblastoma protein (Rb) that attenuates progression of oncogenic Kras-induced carcinogenesis in the pancreas by mediating the senescence response and promoting activity of the tumor suppressor p53 [35]. Phosphorylation of CrkII on Y221 serves as an autoinhibitory protein to negatively regulate tyrosine kinase and decreases fibroblast migration [36]. Fibroblast Growth Factor Receptor Substrate 2 (FRS2) [37] can serve as a pro- and anti-fibrotic protein. When phosphorylated, FRS2 serves as a negative feedback mechanism to regulate and attenuate FGF-induced signaling, and this mechanism prevents pro-fibrotic signaling. Phosphorylation of the Forkhead Box O1 (FOXO1) and O3 (FOXO3) transcription factors at positions T24 and T23, respectively, renders these proteins to act as tumor suppressors and play a role in regulating fibrosis. Additionally, FOXO1 and FOXO3 promote cell cycle arrest and induce apoptosis [38]. Enolase-2 was increased in PSCs with CCK-BR-KO and has recently been found to promote ferroptosis and inhibit glycolysis in clear cell renal cell carcinoma by regulating Hippo-YAP1 signaling [39]. Although sometimes associated with pro-fibrotic activity, emerging roles of PLCγ1 have been described in endothelial biology and the regulation of VEGF [40]. Overall, the RPPA protein analysis supports the RNA sequencing results showing that downregulation of the CCK-BR on PSCs decreases proliferation, migration, and EMT and increases apoptosis and antifibrotic pathways.

Expression of several collagen genes was downregulated in CCK-BR-KO PSC compared to wild-type cells by RNA sequencing. Therefore, we utilized western blot analysis to examine the protein expression of two fibrosis-associated proteins (collagen1α1 and alpha smooth muscle actin (αSMA)) in wild-type and CCK-BR-KO PSC grown independently (Figure 5C, left) or in co-culture with pancreatic cancer cells (Figure 5C, right). Analysis by densitometry of the western blot showed that collagen1α1 expression was decreased in PSC lacking the CCK-BR, and this reduction was more pronounced by co-culture with the cancer cells. CCK-BR-KO did not alter αSMA protein expression of PSCs when cultured independently (Figure 5E, left); however, a pronounced change in αSMA protein expression was observed between the wild-type and knockout cells with pancreatic cancer cell co-culture (Figure 5E, right).

### 2.5. CCK-BR Pharmacologic Blockade in Human PSC (hPSCs) with Proglumide

**Figure 6:**
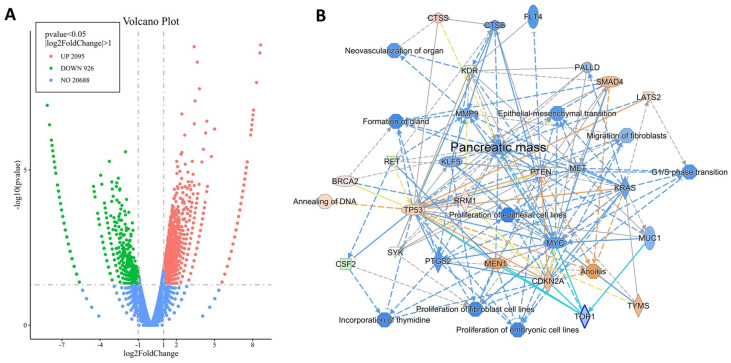
Differentially expressed genes (DEGs) found between untreated and proglumide-treated human pancreatic stellate cells. (**A**) Volcano plot showing 926 downregulated genes (green) and 2095 upregulated genes (orange) comparing untreated human PSCs to proglumide-treated PSCs. (**B**) Pancreatic cancer pathway analysis networks show that genes involved in proliferative and metastatic pathways in pancreatic cancer are downregulated by proglumide (shown in blue), and tumor suppressor genes and genes that are involved with apoptosis are upregulated with proglumide treatment of hPSC (shown in orange). Solid lines imply a direct interaction and dashed lines imply an indirect interaction.

To evaluate the pertinent clinical relevance of the findings observed when the CCK-BR was downregulated by gene editing in murine PSCs, we studied the effects of pharmacologic blockade of the CCK-BR with the CCK-BR antagonist proglumide in human PSCs. The untreated or proglumide-treated human PSC subjected to RNA sequencing found significant changes in differentially expressed genes. A volcano plot shows 2095 genes were upregulated and 926 genes were downregulated in human PSCs treated with proglumide (Figure 6A). IPA pathway analysis demonstrated that the pathways involved in pancreatic cancer that were downregulated by proglumide were similar to the CCK-BR-KO murine PSCs, including pathways that decrease migration, proliferation, and epithelial-to-mesenchymal transition. Specific genes identified that were significantly downregulated in hPSCs by proglumide are shown in Figure 6B in blue color and include genes in cancer proliferation pathways (*KRAS*, *MYC*, *MET*). Certain genes that are upregulated include the tumor suppressor genes (*TP53*, *CDKN2A*, *SMAD4*, *LATS2*, *PTEN*) (Figure 6B; orange color).

### 2.6. Common Genes in Mouse and Human PSCs That Are Affected by Interference of CCK-BR Signaling

**Figure 7:**
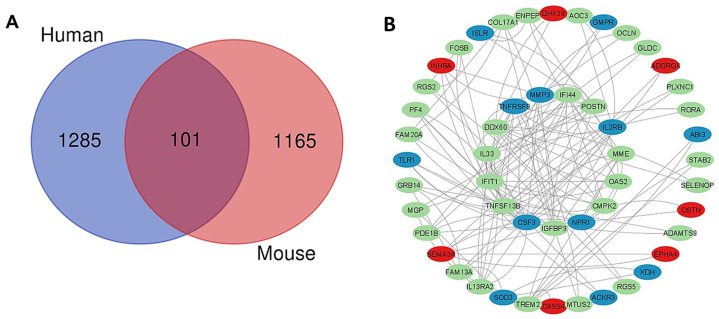
(**A**) A Venn intersection diagram was used to identify 101 common differentially expressed genes (DEGs) between human (in blue) and mouse (in red) datasets (*p* < 0.05 and |logFC| > 1). (**B**) Protein–protein interaction (PPI) analysis was performed to explore functional associations. The PPI network was constructed using the STRING database (https://string-db.org/) and subsequently visualized and analyzed in Cytoscape (https://cytoscape.org/ version 3.9.1).

A comparison was made between the DEGs by RNAseq in the mouse and human PSC for similarities. Analysis shows that there were 101 common DEGs between the CCK-BR-KO mouse PSCs and the proglumide-treated human PSCs (Figure 7A), and their protein–protein interactions are shown in the Cytoscape image (Figure 7B). Most of the common genes that were downregulated and involved in cancer growth, progression, and metastasis are shown in Table 1. The published reference supporting the gene’s role in cancer or fibrotic conditions is shown next to the function. The most downregulated gene in the human PSC by proglumide that was also decreased in the mouse PSC was CDH23, or Cadherin 23. High levels of CDH23 in pancreatic cancer patients are associated with shorter overall survival and correlated with local recurrence and distant metastasis [41]. These similarities serve to validate the murine PSC model and support that the interruption of the cross-communication between activated mouse PSC and pancreatic cancer cells is similar in human tissues. The genes that were not in common may be due to species differences or the possibility that proglumide has other mechanisms of action on PSCs that are not mediated via the CCK-BR signaling pathway.

## 3. Discussion

In this investigation, we studied the molecular pathways involved in the CCK-B receptor signaling pathway of pancreatic stellate cells and how this signaling pathway is important for the communication between the PSCs and the pancreatic cancer cells of the tumor microenvironment. Although other researchers have described the cross-communication between PSCs and pancreatic cancer cells [53], this work is unique because the focus is on the importance of the CCK-BR signaling pathway mediating this communication. We hypothesized that interference with the CCK-BR signaling pathway would shift PSCs to a more quiescent phenotype and reduce their pro-fibrotic and tumor-supportive activity to decrease the growth of pancreatic cancer. Indeed, we showed phenotypic changes in the functions of the PSCs with CRISPR knockout of the CCK-BR, with decreased migration and proliferation, as well as decreased production of collagen1α1 and αSMA, components of the extracellular matrix. In particular, we showed that PSCs communicate with pancreatic cancer cells to promote growth and activate genes involved with cancer invasion and metastases. Interruption of this pathway with CRISPR knockout of the CCK-BR in pancreatic stellate cells significantly reduces the growth of cancer cells as well as the migration and proliferation of the fibroblasts. Some mechanisms involved with cell-to-cell communication are the release of metabolites, chemokines, proteins, and extracellular vesicles carrying cargo such as microRNAs [8,54]. Using the transwell cell culture technique and application of conditioned media to pancreatic cancer cells from PSCs, we confirmed that the CCK-BR pathway is involved in the cross-communication between the cancer cells and the PSCs.

One reason for the poor response of pancreatic cancer is that this cancer is considered a “cold tumor” and lacks CD8+ tumor-infiltrating lymphocytes. Therefore, the use of immune checkpoint antibodies has largely failed in PDAC [55]. The dense fibrosis of PDAC’s tumor microenvironment (TME) is another reason why immune checkpoint antibodies and chemotherapeutic agents are less effective in this cancer type [56]. The pancreatic cancer TME prevents permeation of many agents and penetration of effector T-killer lymphocytes [57,58]. It is well known that the pancreatic tumor microenvironment (TME) plays an important role in pancreatic cancer progression and invasion. CCK-BRs have also been identified in tissue fibroblasts [59] and pancreatic stellate cells (PSC) [60]. When CCK-BRs are stimulated, the PSCs become activated and produce collagen, the major component of the fibrosis in chronic pancreatitis and pancreatic cancer [61,62]. We demonstrated that CCK receptors play an important role in the dense fibrosis of the pancreas TME; treatment of p48-Cre/LSL-Kras^G12D^ (KC) mice with proglumide decreased fibrosis in the TME [63]. We previously showed that murine and human-activated pancreatic fibroblasts treated in vitro with proglumide decreased the migration and proliferation of stellate cells [17]. In the current investigation, we confirmed that this effect was mediated by the CCK-BR since CRISPR knockout of the CCK-BR in PSC also blocked the migration and proliferation. In addition, PSC and pancreatic cancer cells communicate with each other to promote cancer growth and metastases.

Anti-fibrosis strategies have been attempted with agents that inhibit the activation or function of stellate cells [64] or with anti-cytokine [65] therapies. Unfortunately, many of these compounds are not specific to the fibroblasts and may adversely affect other organs. Several methods have been tried to decrease the fibrosis associated with the pancreatic cancer tumor microenvironment [66,67,68,69] in an attempt to improve treatment options. Investigators also studied the use of hyaluronidase to decrease tumor-associated fibrosis and improve chemotherapy [70]. Unfortunately, PEGylated recombinant human hyaluronidase (PEGPH20) combined with nab-paclitaxel and gemcitabine, compared to chemotherapy alone, recently failed in the HALO-301 clinical trial, and the PEGPH20 arm was associated with a significant increase in toxicity [71].

In this investigation, we showed that by blocking the CCK-BR signaling pathway, the mRNA expression of specific genes involved in increased collagen deposition in the extracellular matrix, cancer cell proliferation, invasion, and metastases was significantly decreased. Alternatively, tumor suppressor genes and genes that promote apoptosis were significantly elevated. Interruption of the CCK-BR signaling pathway to induce apoptosis of activated PSCs is indeed a novel approach. Indeed, we showed that cellular markers and molecular markers of apoptosis are significantly increased in the PSCs without the CCK-BR. The same pathways that were affected with the interruption of CCK-BR signaling in the mouse pancreatic stellate cells were also confirmed to be altered by RNA sequencing in human PSCs. Most importantly, we showed that the genes regulating these key cancer pathways in the PSCs were confirmed by examining protein expression with Reverse Phase Protein Array. RPPA is a high-throughput miniaturized calibrated dot-blot immunoassay where hundreds of denatured cell lysates are immobilized onto a nitrocellulose-coated slide using a robotic system. Cell lysates generated from the treated or knockdown stellate cells are immobilized and directly probed with validated primary antibodies targeting unmodified or post-translationally modified residues of the proteins of interest [72]. Specific forms of cellular proteins, including phosphorylated, unmodified, or cleaved residues, can be detected on the RPPA, providing a critical means for broad-scale cell signaling analysis from tissue samples and cell lysates.

Since gene therapy is complicated and expensive in human subjects, finding a small molecule or drug that can mimic the effects of gene knockdown is important. Molecular profiling of the human pancreatic stellate cells after treatment with proglumide, demonstrated that the same pathways were downregulated with CRISPR-*Cas9* that promote pancreatic cancer growth and metastases in the murine model were the same in human PSCs treated with proglumide. These results suggest that the CCK-BR is an oncogene when activated, and therefore suppressing this gene has clinical potential in cancer therapy. The CCK-BR antagonist, proglumide, has oral bioavailability and a broad safety profile in human studies. Due to its ability to remodel the TME, downregulate oncogenic signaling pathways, and interrupt the cross-communication of PSC with cancer cells, proglumide is currently being studied in the clinic as an adjunct treatment for pancreatic cancer (NCT05827055). The anti-fibrotic effects of proglumide have also been evaluated in other conditions, including metabolic dysfunction-associated steatohepatitis [73], and in patients with chronic pancreatitis [74]. Safe and effective strategies to decrease the activity of the PSC without destroying these resident pancreatic cells may lead to improved treatments of chronic pancreatitis and pancreatic cancer.

## 4. Materials and Methods

### 4.1. Cell Lines and Culture Conditions

The mT3-2D (mT3) mouse pancreatic cancer cell line was a gift from Dr. Tuveson’s laboratory and was developed from organoids isolated from mutant *Kras^+/LSL-G12D^*; *Pdx1-Cre* mouse pancreatic cancer lesions [75]. The cells were analyzed by IMPACT II PCR Profile prior to use and found to be pathogen-free. This cell line was previously characterized and found to have CCK-B receptors [76].

Murine pancreatic stellate cells (mPSCs) were isolated from C57BL/6 mice and received as a gift from Dr. Mathison’s laboratory [77]. These mPSCs had been immortalized with SV40 large T-antigen. The mPSCs were cultured in complete DMEM (Gibco, Cat#12491), supplemented with 10% FBS, 1% L-Glutamine, and 1% pen/strep. Primary human pancreatic stellate cells (hPSC) were purchased (Cat#3830; ScienCell Research Laboratories, Carlsbad, CA, USA), and cells were grown in RPMI (Cat#11875093; Gibco, Life Technologies Corporation, Carlsbad, CA, USA) medium supplemented with 10% fetal bovine serum (FBS, Invitrogen, Carlsbad, CA, USA). These cells are negative for HIV-1, HBV, HCV, mycoplasma, bacteria, yeast, and fungi. All cells were grown in vitro with 5% CO_2_ and humidified air.

### 4.2. Downregulation of the CCK-BR with CRISPR Technology and Confirmation by PCR

Murine PSCs were subjected to gene editing using CRISPR-*Cas9* technology and a CCK-BR CRISPR Guide RNA or crRNA1 (Cat#SC1678; GeneScript, Piscataway, NJ, USA). Cells with CRISPR knockdown were selected with puromycin antibiotic (1 µg/mL; Cat# A11138-03 Gibco, ThermoFisher, Waltham, MA, USA). Confirmation of successful CCK-BR mRNA downregulation was determined by real-time PCR (qRT-PCR) using SYBR^®^ Green PCR Master Mix (Cat#4309155; Applied Biosystems, Foster City, CA, USA) in an Applied Biosystems 7300 thermal cycler with the following conditions: initial incubation for 10 min at 95 °C followed by 40 cycles of 95 °C × 30 s, 60 °C × 1 min, and 72 °C for 30 s. CCK-BR primers included (forward) 5′GATGGCTGCTACGTGCAACT3′ and (reverse) 5′CGCACCACCCGCTTCTTAG3′. Murine HPRT was used as a reference gene with PCR primers 5′TCAGTCAACGGGGGACATAAA3′ (forward) and 5′GGGGCTTACTGCTTA-ACCAG3′ (reverse).

### 4.3. Migration Assay

Wild-type and CCKBR-KO mouse PSCs were plated at a density of 10^5^ cells per well in 12-well plates (Cat#1112; PakGent Biosciences, Suzhou, China) and grown to confluency over 48 h. A scratch was created by scratching with a 20 µL sterile tip in each well, and images were taken at 0, 2, 4, 6, and 8 h. The average scratch width from each image was measured in pixels using ImageJ version 1.54.

### 4.4. Proliferation Assay

The 3-(4,5-dimethylthiazol-2-yl)-2,5-diphenyltetrazolium bromide (MTT; Cat# M6494, Invitrogen, Eugene, OR, USA) assay was used to evaluate cell proliferation. Cells were seeded at a density of 10^4^ cells per well in a 96-well plate, 4, 6, and 8 h before the addition of MTT reagent (0.1 mg/mL). After incubating at 37 °C for 2 h, formazan crystals were dissolved with DMSO, and absorbance was measured at 570 nm.

### 4.5. Crosstalk Cell Counts with Murine PSC and Pancreatic Cancer Cells

Cells were seeded at a density of 2.5 × 10^4^ cells per well in 12-well plates. The co-culture setup consisted of PSCs seeded in wells and mT3 cells seeded in the inserts at 5 × 10^5^ cells per insert. The ratio of mT3 and PSCs is 20:1. Cell counts were taken after 5 days of exposure using an EVE automatic cell counter (NanoEnTek; Seoul, Republic of Korea). In a second set of experiments, mT3 pancreatic cancer cells were seeded at a density of 2.0 × 10^4^ cells per well and divided into 3 groups: the first group was cultured with standard DMEM culture media with supplements as above; the second group was cultured with conditioned media from wild-type PSCs; and the third group was cultured with conditioned media from CCK-BR-KO PSC. After 72 h, the mT3 viable cells were counted in the automatic counter.

### 4.6. Western Immunoblotting

Protein extracts (30 µg) from wild-type or CCKBR-KO PSC were loaded on the gel and subjected to electrophoresis with a Pre-stained Plus Protein Ladder (Cat#PM8020, IntelixBio, Beltsville, MD, USA). After protein transfer, the blot was probed overnight at 4 °C with the following antibodies: collagenα1α antibody (titer 1:1000; Cat#PA5-89281; Invitrogen, Carlsbad, CA, USA), αSMA antibody (titer 1:1000; Cat#Ab5694, Abcam, Walton, MA, USA), and GAPDH antibody as the normalizer (titer 1:1000; Cat#Ab8245; Abcam). After washing, the blot was incubated at room temperature with rabbit HRP-linked secondary antibody (titer 1:5000, Cat#31460, Invitrogen). Bands were analyzed by densitometry.

### 4.7. RNA Sequencing

Human PSCs were treated in culture for 48 h with proglumide (20 nM) (COSMA, Bergamo, Italy) or with untreated medium, and total RNA was extracted using the miRNeast Mini Kit (Cat#1038703; Qiagen, Germantown, MD, USA) from cell pellets in triplicate for RNA sequencing. Total RNA was extracted from wild-type and CCK-BR-KO murine PSC (Qiagen; Cat#1038703). RNA integrity was assessed by TapeStation/Bioanalyzer; only samples with RIN ≥ 9 were processed for library preparation and RNA sequencing. Library preparation and sequencing: Strand-specific libraries were prepared by Novogene (Sacramento, CA, USA) using rRNA depletion, and sequenced on Illumina NovaSeq 6000 with paired-end reads (≥PE100; target depth ≥ 30–50 million reads/sample).

Raw sequencing data were processed using Novogene’s standard RNA-seq bioinformatics pipeline. Low-quality reads and adapter sequences were removed with in-house Perl scripts to obtain high-quality clean reads. Clean reads were aligned to the reference genome using HISAT2 (v2.0.5), and gene-level counts were quantified with featureCounts (v1.5.0-p3).

For samples with biological replicates, differential expression analysis was performed using the DESeq2 R package (v1.20.0) based on a negative binomial distribution model. For datasets without biological replicates, the edgeR R package (v3.22.5) was applied after scaling normalization. In both cases, *p* values were adjusted using the Benjamini–Hochberg method to control the false discovery rate (FDR). Genes with an adjusted *p* value ≤ 0.05 and |log_2_FoldChange| ≥ 1 (fold change ≥ 2) were considered significantly differentially expressed.

In parallel, read quality was also evaluated with FastQC; adapters and low-quality bases were trimmed with Trim Galore (cutadapt). Reads were aligned with STAR (v2.7+) to GRCh38 (human) or GRCm39 (mouse) using GENCODE/Ensembl annotations (record versions). Gene-level counts were obtained with featureCounts (Subread v2.0+). Low-abundance genes were filtered (e.g., CPM ≥ 1 in ≥*n* samples). Batch effects were assessed and corrected (e.g., ARSyNSeq or equivalent), and count data were normalized using TMM (edgeR).

### 4.8. Reverse Phase Protein Array (RPPA)

Cells were lysed as previously described and diluted to a final protein concentration of 500 µg/mL [78]. Cell lysates were then immobilized onto a solid substrate, nitrocellulose-coated glass slides (Oncyte Avid, Grace Bio-Labs, Bend, OR, USA), using an Aushon 2470 arrayer (Quanterix, Billerica, MA, USA). Samples were printed in technical replicates along with reference standards used for internal quality control purposes [79]. Selected arrays were used to determine protein concentration in each sample using Sypro Ruby Protein Blot Stain (Molecular Probes, Eugene, OR, USA) [80], and derived protein amounts were used for normalization purposes. Slides were blocked as previously described [80] and immunostained using an automated Epredia Autostainer 360 system (DA, Breda, The Netherlands). The antibodies used for RPPA and their titers, catalog numbers, and vendors are shown in Supplementary Table S1.

### 4.9. Flow Cytometry

Supernatant and cells were collected from wild-type and CCK-BR-KO PSC after 5 days of growth and stained with Propidium iodide, PI (Cat#P4170; Sigma-Aldrich, Rockville, MD, USA) or FITC Annexin V (Cat#640945 BioLegend, San Diego, CA, USA). Cells were subjected to analysis on a BD LSRFortessa flow cytometer analyzer to determine if cells were undergoing apoptosis.

### 4.10. Statistical Analysis

Statistical analysis and graphing were performed with GraphPad Prism software version 10. Analysis was determined by student’s *t*-test comparing two groups with significance set at *p* < 0.05. RNA-seq datasets were uploaded to Ingenuity Pathway Analysis (IPA) software (Qiagen; version Spring Release, Q1 2025)) to identify canonical pathways, diseases, and biological functions associated with differentially expressed genes.

## 5. Conclusions

Pancreatic cancer cells communicate with other cells in the tumor microenvironment to promote their own growth and metastasis. CCK-BRs are present on activated pancreatic stellate cells or fibroblasts, and interruption of this signaling pathway decreases the genes implicated in epithelial to mesenchymal transition, invasion, and metastases in pancreatic cancer. Proglumide, an oral CCK-BR antagonist, which was shown in this investigation to change the molecular profile of activated human pancreatic stellate cells, is currently in clinical trials (NCT05827055). Inhibition of the CCK-BR signaling pathway in the pancreatic cancer fibroblasts has the potential to improve treatments for patients with pancreatic cancer.

## Figures and Tables

**Table 1:** Common DEGs that are downregulated in both mouse and human PSCs when CCK-BR signaling is blocked.

Downregulated Genes	Human log_2_FoldChange	Mouse log_2_FoldChange	Function
*CDH23*	−6.359513328	−2.558595917	High levels of cadherin 23, CDH23 expression, are correlated with a poor prognosis in pancreatic cancer [41].
*TLR1*	−6.359513328	−2.289812863	Toll-like receptors (TLRs) in the liver compartment have been attributed to the development of fatty liver disease [42].
*ACKR3*	−6.041971044	−2.497630078	Atypical chemokine receptors (ACKRs) [43].
*MPEG1*	−5.634213732	−3.623322785	Macrophage-expressed gene 1 codes for a pore-forming protein [44].
*IL2RB*	−2.971465437	−2.210742642	β chain of the IL-2 receptor. Aggravates fibrosis by macrophage activation [45].
*MMP3*	−2.49685765	−4.049810234	Matrix metalloproteinase-3 promotes fibrosis and cancer development by remodeling the (ECM), stimulating (EMT) [14].
*RASGRP2*	−2.313596925	−2.456959022	Activates small GTPases like Rap1 and R-Ras and increases malignant progression [46].
*PTH1R*	−2.149003603	−1.735983328	Parathyroid hormone receptor 1 (PTH1R or PTHR1) promotes Pancreatic cancer growth and metastasis [47].
*TGM1*	−2.069707582	−2.817073954	Transglutaminase-1 high expression can promote tumor progression, metastasis, and cancer stemness by cross-linking proteins [48].
*MEGF6*	−1.925472016	−3.095780165	Multiple epidermal growth factors: they are linked to promoting tumor growth, metastasis [49].
*TNFRSF9*	−1.72413648	−1.811344963	Tumor necrosis factor family promotes metastasis of pancreatic cancer by regulating M2 polarization of macrophages [50].
*NPR1*	−1.692027853	−2.256071133	Nuclear protein 1 is a human gene that promotes pancreatic cancer and confers drug resistance [51].
*RASGRF1*	−1.563551117	−8.042302297	Encodes the protein Ras-GRF1, a dual-function GEF that activates both Ras and Rac GTPases [52].

## Data Availability

The original data presented in this study are openly available at the following site: DOI: 10.5281/zenodo.17306418.

## Supplementary Materials

The following supporting information can be downloaded at: https://www.mdpi.com/article/10.3390/ijms262311699/s1.

## Abbreviations

The following abbreviations are used in this manuscript:

αSMA	alpha smooth muscle actin
CCK-BR	Cholecystokinin-B receptors
CAFs	cancer-associated fibroblasts
DEGs	differentially expressed genes
ECM	extracellular matrix
EMT	epithelial to mesenchymal transition
IPA	Ingenuity Pathway Analysis
KO	knockout
MMPs	matrix metalloproteinases
MTT	3-(4,5-dimethylthiazol-2-yl)-2,5-diphenyltetrazolium bromide
PDAC	pancreatic ductal adenocarcinoma
PSC	pancreatic stellate cells
qRT-PCR	quantitative reverse transcription polymerase chain reaction
RIN	RNA integrity numbers
RPPA	Reverse Phase Protein Array
TME	tumor microenvironment
WT	wild-type
