# Supplementary material for: Downregulation of the CCK-B Receptor in Pancreatic Stellate Cells Blocks Molecular Proliferative Pathways and Increases Apoptosis to Decrease Pancreatic Cancer Growth In Vitro
Source: Int J Mol Sci. 2025 Dec 3;26(23):11699. doi: 10.3390/ijms262311699 (PMC12691738; doi:10.3390/ijms262311699)
Supplement: Supplementary file 1 [file ijms-26-11699-s001.zip › ijms-3955700-supplementary.pdf]

Antibody	Protein Nomeclature	Company	Cat #	Dilution	Host
AKT (S473) XP	AKT_(S473)	CellSig	4060	1:100	R
B-Raf (S445)	B_Raf_(S445)	CellSig	2696	1:50	R
Catenin (beta) (S33/37/T41)	Catenin_B_(S33_37_T41)	CellSig	9561	1:100	R
CrkII (Y221)	CrkII_(Y221)	CellSig	3491	1:100	R
DCLK1	DCAMKL1	Abcam	AB31704	1:200	R
EGFR (Y1148)	EGFR_(Y1148)	ThermoFish	44-792G	1:100	R
Enolase-2 (E2H9X) XP	Enolase-2_Total_[E2H9X]	CellSig	24330	1:100	RmAb
ERK 1/2 (T202/Y204)	ERK_1_2_(T202_Y204)	CellSig	9101	1:1000	R
FKHR-FOX01 (T24)/FKHRL1-FOX03 (T32)	FKHR_FOX01_(T24)_ FKHRL1_FOX03_(T32)	CellSig	9464	1:200	R
FRS2-alpha (Y436)	FRS2_alpha_(Y436)	CellSig	3861	1:50	R
GSK-3a/B (S21/9)	GSK_3a_B_(S21_9)	CellSig	9331	1:100	R
Lck (Y505)	Lck_(Y505)	ThermoFish	44-850G	1:50	R
mTOR (S2448)	mTOR_(S2448)	CellSig	2971	1:100	R
NCAM1 (CD56) (E7X9M)	NCAM1_CD56_Total_[E7X9M]	CellSig	3590	1:2000	R
PKA C (T197)	PKA_C_(T197)	CellSig	4781	1:200	R
PKC a/BII (T638/641)	PKC_a_BII_(T638_641)	CellSig	9375	1:100	R
PKC zeta/lambda (T410/403)	PKC_zeta_lambda_(T410_403)	CellSig	9378	1:50	R
PLC-gamma-1	PLCgamma1_Total	CellSig	2822	1:500	R
Rb (S780)	Rb_(S780)_[C84F6]	CellSig	3590	1:2000	R
Snail (SN9H2)	Snail_Total_[SN9H2]	CellSig	4719	1:2000	Rat
Src (Y527)	Src_(Y527)	CellSig	2105	1:200	R
Stat2 (Y690)	Stat2_(Y690)	CellSig	4441	1:100	R
Vimentin (D21H3)	Vimentin_Total_[D21H3]	CellSig	5741	1:200	RmAb
Wnt5a/B (C27E8)	Wnt5a/B_Total_[C27E8]	CellSig	2530	1:100	RmAb
YAP (S127) (D9W2I)	YAP_(S127)_[D9W2I]	CellSig	13008	1:100	RmAb
**R: rabbit polyclonal; RmAb: rabbit monoclonal**					

Supplementary Table S1. Antibody list for RPPA
